# Bioconcentration, maternal transfer, and toxicokinetics of PFOS in a multi-generational zebrafish exposure

**DOI:** 10.1093/etojnl/vgae033

**Published:** 2025-01-06

**Authors:** Kurt A Gust, Ashley N Kimble, J Erik Mylroie, Michael L Mayo, Mitch S Wilbanks, Catherine S C Steward, Kacy A Chapman, Guilherme R Lotufo, Natalia Garcia-Reyero, David W Moore

**Affiliations:** US Army, Engineer Research and Development Center, Environmental Laboratory, Vicksburg, Mississippi, United States; US Army, Engineer Research and Development Center, Environmental Laboratory, Vicksburg, Mississippi, United States; US Army, Engineer Research and Development Center, Environmental Laboratory, Vicksburg, Mississippi, United States; US Army, Engineer Research and Development Center, Environmental Laboratory, Vicksburg, Mississippi, United States; US Army, Engineer Research and Development Center, Environmental Laboratory, Vicksburg, Mississippi, United States; Bennett Aerospace, Cary, North Carolina, United States; US Army, Engineer Research and Development Center, Environmental Laboratory, Vicksburg, Mississippi, United States; US Army, Engineer Research and Development Center, Environmental Laboratory, Vicksburg, Mississippi, United States; US Army, Engineer Research and Development Center, Environmental Laboratory, Vicksburg, Mississippi, United States; US Army, Engineer Research and Development Center, Environmental Laboratory, Vicksburg, Mississippi, United States

**Keywords:** zebrafish, perfluorooctane sulfonic acid, bioconcentration factor, maternal transfer, toxicokinetic modeling

## Abstract

To enable risk characterization of perfluorooctane sulfonic acid (PFOS) in extended chronic and multi-generational exposures, we assessed PFOS bioconcentration in zebrafish (*Danio rerio*) exposed continuously to environmentally-relevant PFOS concentrations (0, 0.1, 0.6, 3.2, 20, and 100 µg/L PFOS) through 180 days postfertilization (dpf) in parental (P) and first filial generation (F1) fish. Exposures included five replicate tanks per treatment where whole-body PFOS concentrations were measured using 20–35 fish per replicate at 14 and 29 dpf in the P generation and one fish of each sex per replicate at 180 dpf for the P and F1 generations. Perfluorooctane sulfonic acid accumulation reached an apparent steady state at ≤ 14 dpf where whole-body wet-weight concentrations remained constant through 180 dpf in the P and F1 generations. The median bioconcentration factor (BCF) of 934 L/kg was observed for all PFOS exposures with a range from 255 to 2,136 L/kg which varied with PFOS exposure concentration and sex of adult fish. Significantly lower BCFs were observed in 20 and 100 µg/L PFOS exposures versus 0.1 and 0.6 µg/L indicating exposure-concentration dependance. Additionally, males had significantly increased (∼2×) PFOS accumulation and BCFs versus females in both P and F1 generations. Maternal transfer of PFOS was observed from P females to F1 eggs where maternal whole-body and egg PFOS burdens were equivalent, suggesting PFOS transfer to eggs was not a depuration pathway. Finally, a toxicokinetic model was developed that reliably reproduced PFOS whole-body burdens (data within 1.64-fold of predicted values) across all exposure durations spanning the P and F1 generations, providing a tool for PFOS bioaccumulation predictions relevant for risk assessment of acute, chronic, and multi-generational exposures.

## Introduction

The presence of per- and polyfluoroalkyl substances (PFAS) in the environment pose exposure risks to diverse ecological systems and to humans. A recent review evaluating the state-of-the-science for ecological risk characterization of PFAS identified the need for measuring and predicting PFAS bioaccumulation as a significant challenge and a critical need for understanding effects on ecological and human health (Ankley et al., 2021). One such PFAS, perfluorooctane sulfonic acid (PFOS), represents a broadly distributed environmental contaminant in surface waters (Podder et al., 2021) which is toxic to a variety of aquatic organisms including fish (Beach et al., 2006; Gust et al., 2024; Krupa et al., 2022; Lee et al., 2020; Pandelides et al., 2024). Evaluation of PFAS accumulation and bioconcentration data summarized for aquatic species indicated the potential for substantial bioconcentration of PFOS where the median concentration in fish tissues was three orders of magnitude higher than the exposure water concentration (Burkhard, 2021). Although PFOS bioconcentration has been broadly documented in fish, bioconcentration factors (BCFs) may vary greatly even within individual species, as illustrated by Tal and Vogs (2021), where BCF values summarized for zebrafish whole-body tissues ranged from 82 to 5,400, varying with exposure concentrations, exposure durations, and developmental life stages.

In this study, we sought to determine how variables such as exposure concentrations, exposure durations, and developmental life stages affected PFOS accumulation in zebrafish. Specifically, we investigated PFOS accumulation and bioconcentration in zebrafish whole-body tissues resulting from continuous exposure of embryos to a range of environmentally relevant target PFOS concentrations (0 [control], 0.1, 0.6, 3.2, 20, 100 µg/L PFOS, nominal, note: measured concentrations used in analyses) through larval (14 dpf), juvenile (29 dpf), and adult (180 dpf) developmental stages in the parental generation (P) and in continuous exposures persisting through 180 dpf in the subsequent first filial generation (F1). Zebrafish tissues were collected during the multigenerational PFOS exposure study described in Gust et al. (2024), providing direct read-across to ecotoxicological observations described therein, where the threshold for adverse effects occurred at the highest exposure concentration (100 µg/L). A previous long-term (5 months) PFOS exposure in zebrafish indicated the potential for male zebrafish to accumulate greater whole-body burdens than females (Wang et al., 2011), therefore we comparatively evaluated PFOS accumulation among sexes in the P and F1 adults. Additionally, because maternal transfer of PFOS to eggs has been demonstrated in salmonid species (Conard et al., 2022), catfish (Wang et al., 2023), and zebrafish (Sharpe et al., 2010), we evaluated whether maternal transfer of PFOS from P generation females to the F1 generation eggs could serve as a pathway for PFOS depuration and explain the observed decreased female body burdens relative to males. We tested a series of null-hypotheses that PFOS exposure concentration, PFOS exposure duration, zebrafish developmental stage, and sex of the zebrafish had no effect on PFOS accumulation in whole-body tissues or BCFs; and, regarding maternal transfer of PFOS, that there was no difference in zebrafish P generation maternal whole-body PFOS concentrations versus PFOS concentrations in F1 eggs.

In addition to these experimental investigations, we adapted the toxicokinetics model of Warner et al. (2022) to better understand how PFOS accumulation depends on experimental variables such as exposure concentration and duration across multiple zebrafish life stages in a multi-generational PFOS exposure dataset. Our approach differed from other zebrafish toxicokinetics models by accounting for exposure across the entirety of the zebrafish growth and development cycle, including embryo development, larval hatching (between 48 and 80 hr postfertilization [hpf]), and growth into reproductively mature adults. A key contribution of this model is that it can be used to simulate the type of environmentally relevant multigenerational exposure datasets reported here, and the model reliably reproduces many features of the observed dataset. Overall, this approach provides quantitative support to PFOS environmental risk assessments wherein uncertain exposure conditions create a need to evaluate BCFs across multigenerational timescales and ultimately supports the critical need identified by Ankley et al. (2021) for improved prediction of PFAS bioaccumulation.

## Materials and methods

### Study overview

Zebrafish tissues were collected as part of a multigenerational PFOS exposure described in Gust et al. (2024) as well as an initial launch of that experiment that was ultimately terminated early due to laboratory closure in March, 2020, resulting from the COVID-19 pandemic. Zebrafish were exposed to PFOS at target concentrations of 0 (control), 0.1, 0.6, 3.2, 20 and 100 µg/L that included five replicate exposure tanks with 50 fish per replicate. The initial launch of the experiment provided zebrafish exposed to the experimental treatments from 0 to 14 dpf and 0 to 29 dpf, whereas the complete multigenerational exposure described in Gust et al. (2024) provided zebrafish exposed from 0 to 180 dpf in the P generation as well as zebrafish exposed from 0 to 180 dpf in the F1 generation. Fish within each exposure tank were maintained separately from all other replicates throughout the complete duration of all experiments and throughout the multigenerational exposure to maintain independence. All animal testing was conducted using methods and protocols approved by the US Army, Engineer Research and Development Center’s Institutional Animal Care and Use Committee (IACUC Protocol # EL-6008-2020-1).

### Fish exposures

For a detailed description of the zebrafish exposure methods for the multigenerational exposure, refer to Gust et al. (2024). The following provides a brief overview of the exposures and makes note of where methods for the initial launch exposure differ from Gust et al. (2024). For both the initial launch exposure and the complete multigenerational exposures, Vicksburg, MS (USA) municipal water was used, which was treated by reverse osmosis (RO) followed by filtration through Purofine PFA694 ionic resin (Purolite LLC, King of Prussia, PA) to remove any residual contaminants (including fluorinated chemicals). Heptadecafluorooctanesulfonic acid potassium salt (PFOS; CAS no. 2795-39-3; >98% purity; product #: 77282, lot #: BCCC4690) from Sigma-Aldrich (Saint Louis, MO) was used for all PFOS exposures. Embryos generated from wild-type, AB strain adult zebrafish (Zebrafish International Resource Center, Eugene, OR) were exposed to PFOS beginning at approximately 7 hpf and maintained at 28.5°C with a 14:10-hr light:dark cycle.

For the initial launch exposure, 80 embryos per replicate were exposed in 500 ml of each experimental treatment solution in estradiol (E2) media (Varga, 2016a) in 1 L polycarbonate tanks until 5 dpf. Starting at 5 dpf, the volume in the tanks was raised to 750 ml, zebrafish were fed GEMMA Micro 75 (Skretting USA, Tooele, Utah) twice daily, and 80% water exchanges were conducted once daily. At 15 dpf, 40 zebrafish larvae per replicate were transferred to a ZebTEC Stand-Alone Toxicology Rack (Tecniplast, Buguggiate, Italy) flow-through exposure system with replicates maintained in individually aerated 3 L polycarbonate chambers containing 2.8 L of experimental treatment water, which was exchanged three times daily as described in Gust et al. (2024). After transfer, feeding continued with GEMMA Micro 75 twice daily until exposure termination at 29 dpf. At 14 and 29 dpf, P fish were collected from each of the five replicate tanks and flash frozen for tissue analysis.

In the complete multigenerational exposure (Gust et al., 2024), embryos were housed in 75 mm polypropylene petri plates (Eisco Scientific, Victor, NY, USA) containing 23 ml of each experimental treatment solution (control or PFOS exposures) through 5 dpf, and then sets of 50 zebrafish were transferred to each of five replicate 3 L polypropylene exposure chambers containing approximately 800 ml of each corresponding experimental treatment solution prepared in E2 media. After this transfer to the exposure tanks, the zebrafish were fed a rotifer suspension and GEMMA Micro 75. Static renewal exposures were maintained from 5 to 30 dpf with the exposure solution volume gradually raised to 2 L by 20 dpf and all replicates receiving an 80% water exchange twice daily. At 30 dpf, zebrafish were transferred to a ZebTEC Stand-Alone Toxicology Rack flow-through exposure system with replicates maintained in individually aerated 3 L polycarbonate chambers containing 2.8 L of experimental treatment water that was exchanged three times daily. At 30 dpf, rotifer feeding ceased, and GEMMA Micro 75 feeding increased to three times per day, which was then replaced by feeding with 40 mg GEMMA Micro 150 twice per day at 50 dpf through 111 dpf per replicate. At 111 dpf, the fish within each replicate chamber were reduced to 15 males and 15 females (some replicates deviated from this ratio either due to skewed sex ratios or mortality resulting in fewer than 30 fish at Day 111) to establish an even sex ratio and fed a ration of 3.8 mg/fish of GEMMA Micro 500 fish food twice per day through 180 dpf. This fish food was analyzed for PFOS, returning values below the analytical limit of detection (LOD) of 75 ng PFOS/kg (see Gust et al., 2024 for analysis details).

The F1 zebrafish were exposed through 180 dpf using identical methods as the P generation described above. At the completion of each exposure, fish were euthanized by an overdose of buffered 4 g/L tricaine methanesulfonate (MS-222; Sigma-Aldrich; CAS no. 886-86-2; Product #: A5040). Whole fish were collected for PFOS tissue residue analyses at 14 and 29 dpf (30–35 fish per exposure replicate at 14 dpf and 20–25 per replicate for the 29 dpf) and at 180 dpf for both the P and F1 generations where the sexes were analyzed separately (1 male and female fish per exposure replicate). All samples were flash frozen in liquid nitrogen and then stored at −20°C prior to analysis.

### PFOS maternal transfer investigation

To investigate the potential for maternal transfer of PFOS to developing eggs, P generation females exposed through 180 dpf were moved to clean water, spawning was induced, and the F1 generation eggs were collected and the PFOS tissue burdens determined. The complete description of the spawning methods is provided in Gust et al. (2024). A brief description of the breeding methods is provided in the following. At 179 dpf, all males and females from each replicate were divided in a 1:1 ratio between two 1.7 L Slope Breeding Tanks (Tecniplast, Buguggiate, Italy) filled with control water the afternoon before the breeding event. Fish within each replicate chamber from the P generation were bred, keeping all replicates separated. The zebrafish were housed in these chambers overnight with males and females separated by a plastic divider. The next morning (Day 180) the dividers were removed, and the fish were allowed to spawn for approximately 45 minutes. After the spawning period, adult fish were removed and all eggs were collected and counted. A subset of 250 eggs from each breeding tank were surface sanitized, screened for fertilization, and then used for the F1 generation (Gust et al., 2024), and all remaining eggs from each breeding tank were collected in E2 media and housed in petri dishes at 28.5°C. From this set of eggs, two sets of approximately 100 eggs were collected at 7–8 hpf from each replicate and placed in 1.5 ml freezing vials and then the excess E2 media was removed using a micropipette. The aliquots of 100 eggs were then flash frozen using liquid nitrogen and stored at –80°C until analysis.

### Water quality

Water quality measurements for the P 14 and 29 dpf exposures consisted of dissolved oxygen, temperature, conductivity, pH, and salinity, which were measured using a YSI Professional Plus multimeter (Xylem Inc., Washington, DC) and are presented in online supplementary material Table S1. Water quality values for the P and F1 180 dpf exposures included dissolved oxygen, temperature, conductivity, pH, salinity, and total ammonia, which are reported in Gust et al. (2024) and reproduced in online supplementary material Table S2. Water parameter measurements were within acceptable ranges for zebrafish housing conditions, as specified in Varga and Murray (2016b) and the Organisation for Economic Co-operation and Development (OECD) fish sexual development standard test (OECD, 2011).

### Exposure water sampling and PFOS analysis

Perfluorooctane sulfonic acid was measured in exposure water samples taken every 1 to 2 weeks through the complete duration of the zebrafish exposures, following the methods described in Gust et al. (2024). Briefly, 7 ml water was collected from each treatment (control, 0.1, 0.6, 3.2, 20, and 100 µg PFOS/L) in 15 ml polypropylene centrifuge tubes at each sampling time, where two replicate exposure chambers per treatment were sampled and then stored at 4–6°C in the dark until PFOS analysis was conducted. The 7 ml water samples were diluted in the original collection vessel with 7 ml methanol (MeOH) then diluted as needed to fall within the analytical instrument’s linear range for PFOS. In the final dilution, an internal standard was administered at 0.7 µg/L to each sample. Exposure water samples were analyzed by liquid chromatography triple quadrupole tandem mass spectrometry (LC-MS/MS) using an Agilent 1290 Infinity Binary Pump LC coupled to an Agilent 6495B triple quadrupole MS/MS with jet streaming technology and electrospray ionization (ESI). Chromatographic separation was performed using an Agilent Poroshell 120 EC C18column (2.1 × 100 mm, 1.9 µm) using an Agilent Eclipse Plus C18 RRHD column (3.0 × 50 mm, 1.8 µm) between the pump and autosampler to delay any protentional PFOS that is inherently in the system. Data acquisition was performed in dynamic multiple reaction monitoring mode using negative mode ESI. Chromatographic separation was achieved by gradient elution with a flow rate of 0.4 ml/min using 10 mM ammonium acetate with 3% MeOH in LC-MS grade water as mobile phase A and 10 mM ammonium acetate with 20% acetonitrile in MeOH as mobile phase B. The analytical column was held at a temperature of 50°C during separation. The LOD and limit of quantitation (LOQ) for PFOS in exposure water samples was 12 ng/L and 40 ng/L, respectively.

### PFOS extraction from zebrafish whole-body tissue and egg samples and analysis

Perfluorooctane sulfonic acid was extracted from all five replicates to determine zebrafish whole-body tissue concentrations and eggs collected from three randomly selected replicates to establish PFOS maternal transfer. All PFOS accumulation concentrations were calculated based on tissue or egg wet weights. To quantify PFOS in tissue and eggs, samples were digested in (70:30, v/v) acetonitrile: water. Whole fish samples were homogenized in 3 ml extraction solvent using a Pro200 Pro Scientific homogenizer with multi-gen 7XL blades (Pro Scientific, Oxford, CT). Blades were cleaned using acetone, isopropyl alcohol, and acetonitrile and tested for contamination between samples. Egg samples were homogenized in 1 ml extraction solvent using a sonication probe in three intervals of 30 s. After homogenization, samples were sonicated for 4 hr, centrifuged at 2,900 *g* for 15 min, placed in a –20°C freezer overnight, then thawed the following day, centrifuged again, and the final supernatant was filtered through a 0.2 µm nylon filter into a clean polypropylene container for additional sample clean up. Extracts were diluted with methanol as needed before analysis to ensure samples were within the linear range of the instrument for quantitation. National Institute of Standards and Technology–certified reference tissue samples (1947 Lake Michigan fish tissue homogenate, frozen) were also extracted to provide quality control for the tissue analyses. A bile salt interference check standard of taurodeoxycholic acid was monitored at 1 mg/L to ensure that it eluted outside of the window for PFOS and did not result in biased concentrations of PFOS.

Zebrafish tissue samples were analyzed by LC-MS/MS using an Agilent 1290 Infinity Binary Pump LC coupled to an Agilent 6495C triple quadrupole. Chromatographic separation was performed using an Agilent Zorbax Eclipse Plus C18 RRHD column (2.1 × 100 mm, 1.8 µm). Data acquisition was performed in dynamic multiple reaction monitoring mode using negative mode ESI. The analytical methodology was modified from United State Environmental Protection Agency (USEPA) Draft Method 1633 (USEPA, 2021). Chromatographic separation was achieved by gradient elution with a flow rate of 0.35 ml/min using 2 mM ammonium acetate with 5% acetonitrile in LC-MS grade water as mobile phase A and 100% acetonitrile as mobile phase B. The analytical column was held at a temperature of 40°C during separation. The LOD and LOQ for PFOS in tissue samples were 0.6 ng/g and 1.8 ng/g, respectively. However, the concentrations present in tissue and egg samples from exposure tanks were well above LOQ. All analytical chemistry was conducted in-house at the US Army Engineer Research and Development Center.

### PFOS exposure concentrations connected to PFOS tissue burdens

Perfluorooctane sulfonic acid accumulation in zebrafish has been observed to occur rapidly in response to PFOS in exposure water (Tal & Vogs, 2021), whereas PFOS depuration in zebrafish moved to clean water can reduce tissue burdens with a half-life of approximately 9 days (Zou et al., 2021). These observations indicate that tissue concentrations for PFOS in zebrafish vary dynamically with PFOS levels in exposure water, where PFOS burdens are most likely to reflect near-term PFOS exposure concentrations. In our study, both exposure-water samples and tissue samples for PFOS analysis were collected on the same day. Given the potential for PFOS tissue burdens to vary rapidly with near-term PFOS concentrations in the exposure water, we utilized analytical chemistry values taken at the time of each tissue sampling timepoint to attribute PFOS tissue burdens and bioconcentration factor values instead of using the life-time average PFOS exposure values reported in Gust et al. (2024).

### BCF

Bioconcentration factor values were calculated as whole-body tissue wet weight concentrations divided by the mean measured exposure water concentrations sampled on the same date that the fish samples were taken. The methods used for the zebrafish exposures conform to OECD (2012) and USEPA (2016) standard guidelines for determining BCFs where exposures are recommended until steady state is reached or through as many as 28–60 days of exposure. Because bioconcentration tests may persist for extended periods of time, the standard methods prescribe daily feeding as necessary for providing adequate nutrition and maintaining good health. The USEPA (2016) recommends that uneaten food and feces are removed daily from the test vessels shortly after feeding and these recommendations were followed in our study. Although food provided daily may serve as a pathway for uptake of PFOS, the standard guidance documents define bioconcentration as “the net accumulation of a test substance by the fish as a result of uptake directly from aqueous solution, through gill membranes or other external body surfaces” (USEPA, 2016) and do not discuss the potential contribution of food consumed during the standard assays toward measured body residues or provide the means of quantifying such contribution. Because food was provided daily, experimental fish can be assumed to have consumed primarily food that was exposed to PFOS in the water for a very short period of time. Based on the standard guidance documents described above and published studies reporting BCFs for PFAS in fish exposed to contaminated water and fed daily uncontaminated food (e.g., Barber et al., 2023; Huang et al., 2022; Wang et al., 2022; Zhong et al., 2019; and studies reviewed by Burkhard, 2021), we consider that the results presented in our study most accurately represent BCF values for PFOS in zebrafish.

### Statistical analyses

Prior to conducting statistical analyses for PFOS accumulation and BCF, each set of untransformed data was evaluated using Shapiro-Wilk to test whether the dataset was normally distributed (*p *= .05) and, if normally distributed, the Brown-Forsythe test was then used to test whether the dataset had homogeneous variance (*p *= .05). Datasets meeting these assumptions were analyzed using parametric statistical analyses. Analysis of covariance was conducted to investigate the relationships between measured PFOS exposure concentrations and exposure time on PFOS tissue concentrations in zebrafish. One-way analysis of variance (ANOVA) was used to test the effect of exposure time on PFOS accumulation within each PFOS exposure concentration treatment. Two-way ANOVA was used to test for effects of PFOS exposure concentration and exposure time on BCF in the 14 and 29 dpf exposures. For the 180 dpf sampling points, two-way ANOVA was used to test the effects of PFOS exposure level and the sex of the fish on PFOS tissue concentration and BCF individually in the P and F1 generations. Finally, the maternal transfer of PFOS was tested using two-way ANOVA to examine the effects of PFOS exposure concentration and compare PFOS concentrations in the P generation maternal zebrafish and the F1 eggs they produced. Zebrafish whole-tissue analyses included all five replicates, whereas the maternal transfer analysis included three replicates where the egg replicates were paired with their maternal source replicates. Post hoc investigation of all pairwise multiple comparisons was conducted using the Student-Newman-Keuls method for the one-way ANOVA and the Holm-Sidak method for two-way ANOVA. All tests of statistical significance were based on *p *= .05. When datasets failed normality and/or homogeneity of variance tests, the following approaches were used. For datasets where one-way ANOVA was applied, Kruskal-Wallis one-way ANOVA on ranks was conducted and the post hoc analysis methods described above were applied. For datasets where two-way ANOVA was applied, the data were rank transformed and retested using the Shapiro-Wilk and Brown-Forsythe tests, respectively. This transformation method was successful at establishing normality and homogenous variance for noncompliant zebrafish whole-tissue datasets. For the maternal transfer dataset, a log_10_ transformation was applied to meet normality and homogeneity requirements. Once transformed, the parametric two-way ANOVA and post hoc methods described above were applied. All statistical analyses were conducted using SigmaPlot/SigmaStat Ver. 13.0 software (Systat Inc. Palo Alto, CA).

### Toxicokinetic model development

The early development of zebrafish embryo tissues, larval hatching events, and their subsequent growth into adults will affect the maternal transfer of PFOS in zebrafish, because uptake/elimination kinetics and tissue concentrations vary in response to exchange surfaces (e.g., gills) and tissue volumes that grow with time. To account for these growth events, our approach is to extrapolate the multicompartment embryo development model of Warner et al. (2022; chorion, larva, yolk) into a one compartment description of a growing adult zebrafish, which extends its validity beyond the embryo development window of 0–120 hpf. Warner et al. (2022) lumps the perivitelline fluid together with the chorion into a compartment they refer to simply as the “chorion.” This semipermeable chorion compartment mediates PFOS uptake into the growing tissues of the larval compartment, which is treated as a PFOS sink. Uptake of PFOS into larval tissues is limited by the chorion surface area, which remains approximately unchanged as yolk depletes and the larva grows. To extrapolate PFOS uptake by these larvae toward values representative of fish that have developed into reproductively capable adult fish, we need to fill two critical predictive gaps: (1) the increase in tissue volume of adult fish that grow over time and (2) the development of gill exchange surface areas over the 180 dpf period of exposure.

Zebrafish embryos hatch between approximately 48 and 72 hpf, shedding their chorion, which creates a natural separation point between embryo and adult growth regimes. As explained by Warner et al. (2022), the hatching fraction, h(t), is the total number of zebrafish embryos found to have hatched at a time t, which can be modeled with the equation


(1) $$h\left(t\right)=\frac{{\left(\frac{t}{T}\right)}^{\alpha }}{1+{\left(\frac{t}{T}\right)}^{\alpha }}\;.$$

Here, T specifies the time at which half the initial embryo population has hatched (assuming no mortality), and *α* is an exponent related to the maximum rate of hatching. The volume, V(t|s), of an individual zebrafish larva continues to grow over a duration t-s on hatching at a time, s, according to a log-logistic growth function:


(2) $$V\left(t|s\right)=\frac{V_{0}\left(s\right)+V_{\infty }{\left(\frac{t-s}{\tau }\right)}^{2}}{1+{\left(\frac{t-s}{\tau }\right)}^{2}}.$$

Here we calculate the initial volume, $V_{0}(t)$, as the larval volume modeled by Warner et al. (2022); $V_{\infty }$ is a volumetric asymptote, and τ describes a characteristic growth time. Because embryos hatch at different times, we can calculate an average tissue volume, $〈V〉$


(3) $$〈V〉\left(t\right)=\int_{0}^{t}ds\;p\left(s\right)\;V\left(t|s\right),$$

using a probability density function defined by the fraction of hatched embryos, $h\left(t\right)=\int_{0}^{t}ds\;p(s)$.

A countercurrent flow across gill lamellae creates a perfect oxygen sink, and here we assume that PFOS uptake exhibits a similar efficiency such that its overall uptake is proportional to the gill exchange surface area, $S_{\text{gill}}$. Data shows that gill exchange surface areas grow allometrically in proportion to body mass (Scheuffele et al., 2021). A linear regression of these data expressed in a semi-log scale identifies the following relationship between gill surface area and body volume ($R^{2}=0.946$):


(4) $$S_{\text{gill}}=\frac{\rho \;V}{\sigma }.$$

**Figure 1.**
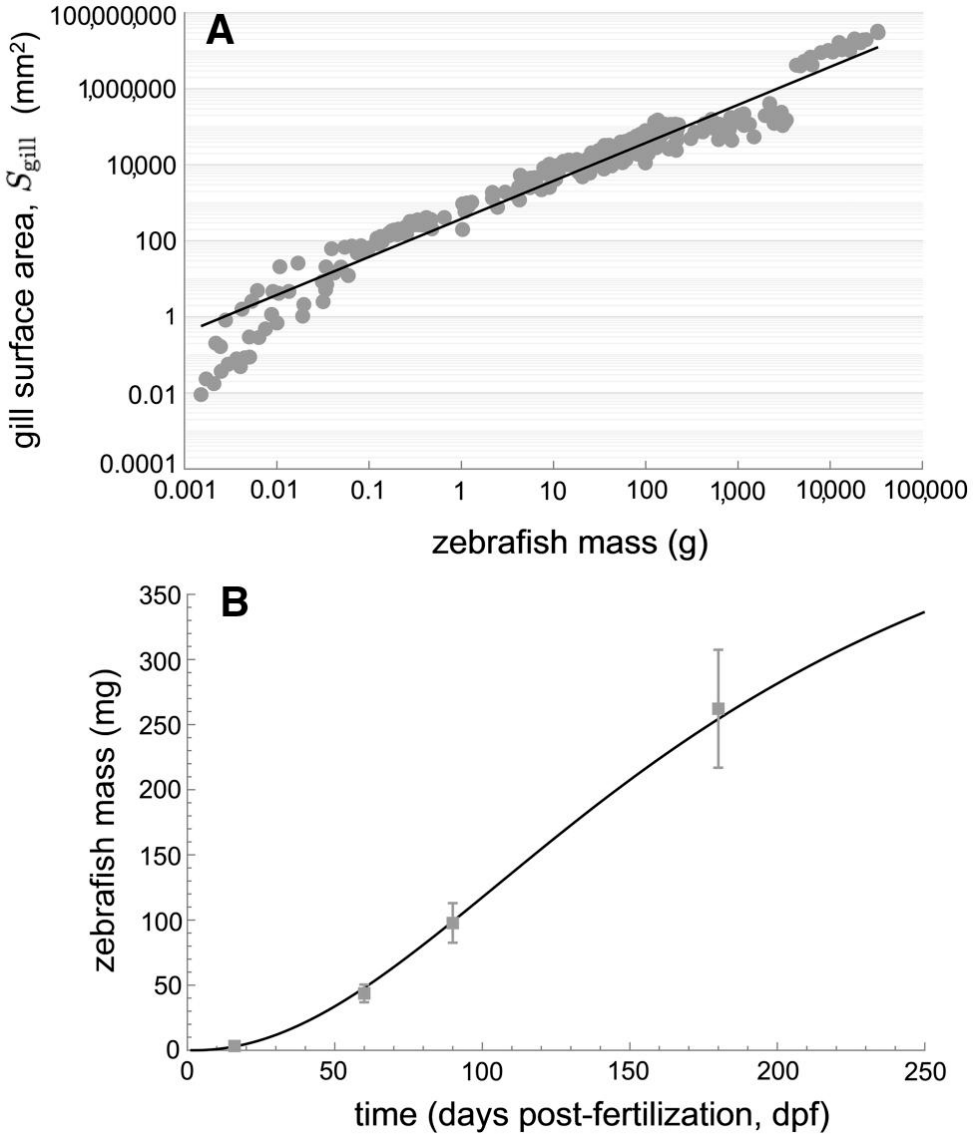
Fitted physiological datasets in support of the toxicokinetic model. Panel (A) illustrates that gill exchange surface areas are allometric, varying with fish body mass (Scheuffele et al., 2021). Panel (B) illustrates the timescale of growth in zebrafish body mass.

Here, ρ=1 g/mm^3^ is the unit density of water, and σ=0.00265 g/mm^2^ is a fitted parameter with units of surface density. Figure 1A illustrates the fidelity of this fit.

We conceptualize the aggregate tissues of a hatched zebrafish larva as a single PFOS reservoir that grows in volume over time and accumulates an amount of PFOS quantified by its concentration C(t). To a reasonable approximation, we assume that PFOS uptake and elimination occurs primarily via transfer over gill surfaces via respiration such that body burden can be modeled using the following rate equation:


(5) $$\frac{d}{dt}\left(V(t)C(t)\right)=kS_{\text{gill}}(t)\frac{C_{\;\text{exp}\;}}{K+C_{\;\text{exp}\;}}-k_{d}S_{\text{gill}}(t)C\left(t\right).$$

The gill surface area (mm^2^) can be calculated using *Equation 4* by expressing *Equation 2* in units of mm^3^, which dynamically scales the uptake and elimination rates over the timescale of fish development. Values for the larval PFOS uptake parameter, k, and its elimination rate, $k_{d}$, are found using a curve fitting approach described below. The parameter K quantifies how the uptake rate depends on the exposure concentration, $C_{\;\text{exp}\;}$. Finally, to compare our model against PFOS data, we should average the individual larval concentrations of *Equation 5* over the hatching density:


(6) $$〈C〉\left(t\right)=\int_{0}^{t}ds\;p\left(s\right)C\left(t-s\right).$$

### Parameter estimation and curve-fitting methodology

Estimation of zebrafish volumes is critical for modeling PFOS tissue concentrations, and we can relate mass data gathered here as part of study to total tissue volumes by assuming that the density of zebrafish tissues is equal to that of water (Robertson et al., 2008). Parameters of mass growth (see Eq. 2) were fit to data using particle swarm optimization (100 particles) to minimize a least squares objective functional weighted to the standard deviation calculated for each mass data point. Figure 1B illustrates the fidelity of this numerical fit.

Because *Equation 5* scales the uptake and elimination fluxes against gill exchange surface areas, existing values for the kinetic rate constants k and $k_{d}$ could not be translated directly from other literature exposure studies using adult zebrafish. Instead, we used the following method to evaluate *Equation 6* for the parental (P) and first filial (F1) whole-organism data gathered as part of this study. Given values for both k and $k_{d}$, we first choose an exposure timepoint, either 14, 29, or 180 dpf. We chose to evaluate *Equation 6* using the hatching distribution discretized over timepoints spanning 48 to 80 hpf in 30-min increments, because outside of this interval the hatching probability is negligible. For a given PFOS exposure timepoint, we simulate a larval concentration for each of these hatching times using the two-compartment model of Warner et al. (2022), using the resulting embryo concentration as initial data for *Equation 5*. To numerically integrate *Equation 6*, we used the trapezoidal rule, which, although it is both fast and computationally efficient, it also generates concentrations that are accurate to approximately 0.01 μM. Parameter values for k and $k_{d}$ are found by using trust-region-reflective methods in MATLAB Ver. 24.1.0.2578822 (MathWorks Inc., 2024) to minimize an unweighted least squares objective functional calculated by first squaring the difference between the logarithm (base 10) of both *Equation 6* and the appropriate data point for all points in the training dataset, and then adding all such contributions together.

## Results

### Summary of exposures

The measured concentrations of PFOS in the exposure water for the P 14 and 29 dpf exposures tended to be greater than the nominal target concentrations by an average of 34% and 59%, respectively (Table 1). Perfluorooctane sulfonic acid concentrations in the exposure water for the P 180 dpf time point were less than the target concentration by an average of 41%, whereas the F1 180 dpf exposure was only slightly less than the target concentrations, on average (Table 1).

**Table 1.** Measured perfluorooctane sulfonic acid (PFOS) concentrations (µg/L) in exposure water at each tissue sampling time point.

14 dpf—Parental (P) generation			
PFOS concentration (µg/L)			
Nominal	Measured mean	Measured *SD*	*n*
Control	<LOD	n/a	2
.1	.12	n/a	1
.6	.7	.1	2
3.2	4.5	.2	2
20	29	0	2
100	143	19	2

29 dpf—P generation			
PFOS concentration (µg/L)			
Nominal	Measured mean	Measured *SD*	*n*
Control	<LOD	n/a	7
.1	.12	.02	7
.6	.8	.2	7
3.2	6	1.4	7
20	36	6	7
100	179	18	7

180 dpf—P generation			
PFOS concentration (µg/L)			
Nominal	Measured mean	Measured *SD*	*n*
Control	<LOD	n/a	2
.1	.05	.01	2
.6	.3	.0	2
3.2	1.3	.1	2
20	14	5	2
100	85	3	2

180 dpf—First filial (F1) generation			
PFOS concentration (µg/L)			
Nominal	Measured mean	Measured *SD*	*n*
Control	<LOD	n/a	2
.1	.08	.01	2
.6	.5	.0	2
3.2	2.9	.1	2
20	25	11	2
100	100	24	2

*Note:* dpf = days postfertilization; n/a = not applicable.

PFOS concentrations are reported as significant figures based on analytical method detection sensitivity. All measurements of control samples returned values below limits of detection (<LOD), which was 12 ng/L. *n* represents the number of replicate samples analyzed for each treatment.

### PFOS accumulation in zebrafish whole-body tissue

**Figure 2.**
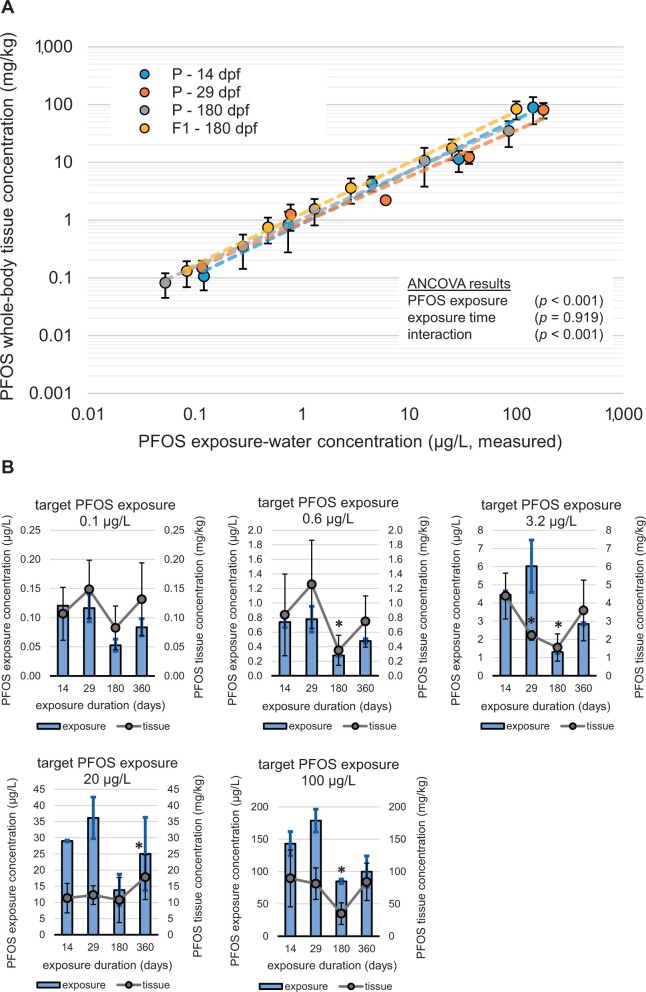
Perfluorooctane sulfonic acid (PFOS) accumulation in zebrafish whole-body tissues based on wet weights. Panel (A) provides PFOS body burden (mg/kg) to exposure concentration (µg/L) comparisons across all exposure durations for parental (P) and first filial (F1) generations analyzed using analysis of covariance. Panel (B) provides PFOS body burdens in response to each exposure water concentration measured for each exposure duration where the 360 day exposure represents the F1 generation exposure after 180 days of parental exposure plus 180 days of individual exposure. Exposures are represented as days postfertilization (dpf). Asterisks represent statistically significant pairwise differences in PFOS concentrations in fish tissue comparing 14 days versus all other exposure durations.

**Figure 3.**
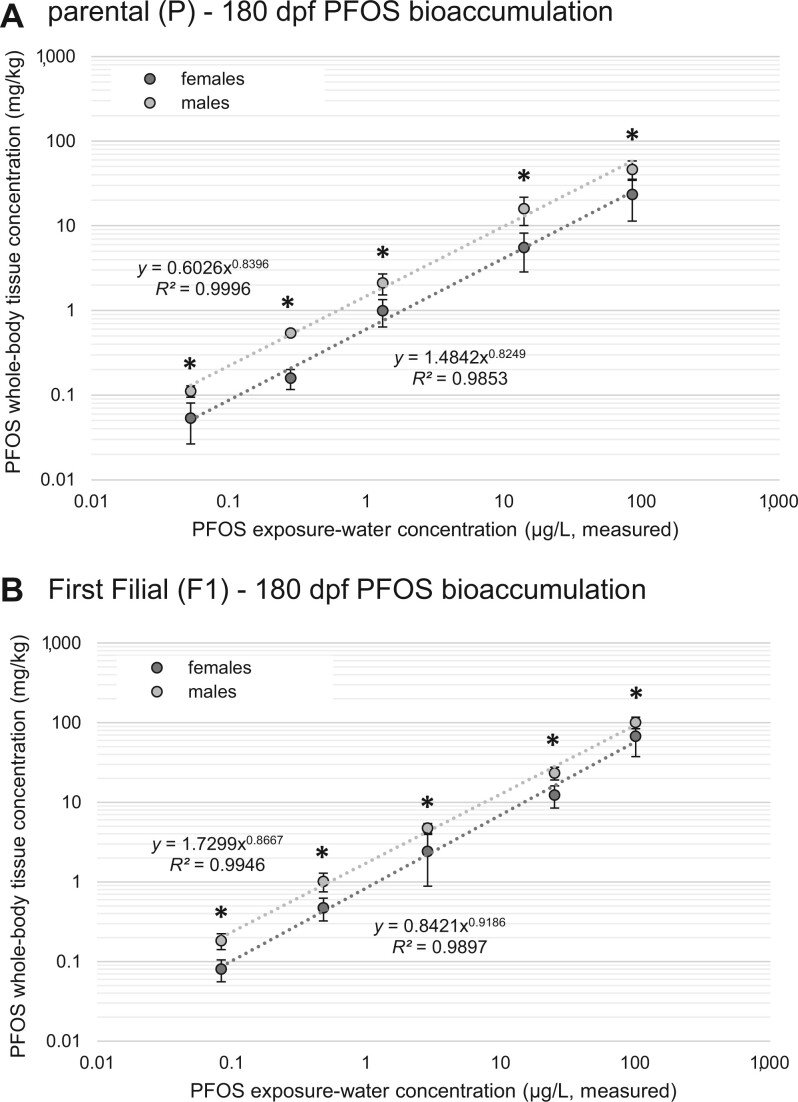
Comparison of perfluorooctane sulfonic acid (PFOS) accumulation among sexes. Values represent PFOS accumulation in zebrafish whole-body tissue wet weights from continuous exposure to PFOS for 180 day postfertilization (dpf) in: (A) the parental (P) generation and (B) the first filial (F1) generation. Asterisks (*) represent significant differences (*p *= .05) among sexes within each exposure concentration.

Perfluorooctane sulfonic acid accumulation in zebrafish whole-body tissues increased significantly with increasing PFOS exposure concentrations (*p *< .001, f = 480), exposure time had no significant effect (*p *< .919, f = 0.166), and a significant interaction (*p *< .001, f = 13.0) was observed between these variables (Figure 2A). Inspection of PFOS concentrations in whole-body tissue within each PFOS exposure concentration (Figure 2B) indicated some significant differences across exposure time; however, there was no consistent pattern across the PFOS concentrations. Overall, these results suggest that PFOS concentrations in zebrafish tissues had likely reached steady state by 14 dpf where the majority of tissue concentrations at subsequent sampling times were either not significantly different or were significantly less than the PFOS concentrations observed at 14 dpf (Figure 2B). Finally, PFOS body burdens were significantly increased in male fish relative to females across all PFOS exposure concentrations after 180 days of exposure in both the P (*p *< .001, f = 61.6) and F1 (*p *< .001, f = 53.3) generations, where average fold-increases in male tissue relative to females were 2.5 and 1.9, respectively (Figure 3).

### PFOS bioconcentration

**Figure 4.**
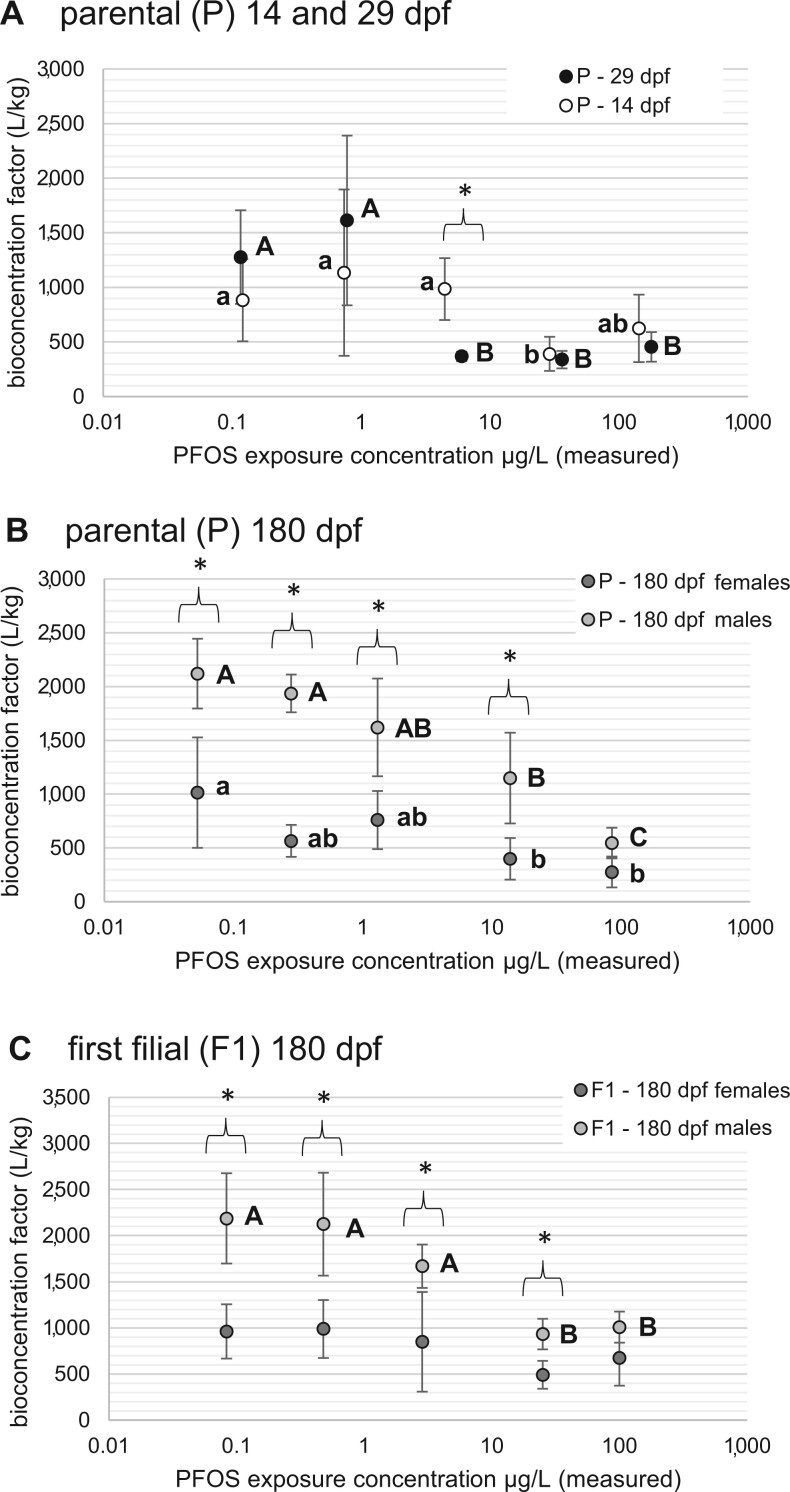
Perfluorooctane sulfonic acid (PFOS) bioconcentration factors (BCF) in zebrafish whole-body tissues based on tissue wet weights. Panel (A) provides PFOS BCFs in parental (P) zebrafish where lowercase letters denote differences in BCF among PFOS exposure concentrations for 14 days postfertilization (dpf) fish and uppercase letters denote BCF differences in 29 dpf fish. Asterisks represent BCF differences between 14 and 29 dpf values at each concentration. Panels (B) and (C) provide PFOS BCFs comparing males and females after 180 days of exposure in the P and first filial (F1) generations, respectively. For panels (B) and (C), lower case letters denote differences in BCFs among PFOS exposure concentration for males and lower-case for females while asterisks represent BCF differences among sexes at each PFOS exposure concentration.

Investigation of PFOS BCFs in the 14 and 29 dpf exposures revealed a trend of significantly decreasing BCF (*p *< .001, f = 14.5) at elevated exposure concentrations relative to the lowest two exposures in sub-µg/L range (Figure 4). With the exception of the intermediate 3.2 µg/L PFOS exposure concentration, no significant differences were observed between BCFs at 14 and 29 dpf. Significant trends of decreasing BCFs with increasing PFOS exposure concentrations were also observed in the 180 dpf time points for both the P (*p *< .001, f = 21.2) and F1 (*p *< .001, f = 12.8) generations (Figure 4). Similar to the observation of increased PFOS accumulation in males, BCF values were significantly increased relative to females for all PFOS exposure concentrations in both the P (*p *< .001, f = 99.2) and F1 (*p *< .001, f = 62.7) generations (Figure 4). Overall, PFOS was strongly bio-accumulative in zebrafish tissues with mean wet weight BCF values, ranging from 276 to 2,187 L/kg with a range for males from to 546 to 2,187 L/kg and 267 to 1,015 L/kg for females.

### PFOS maternal transfer

**Figure 5.**
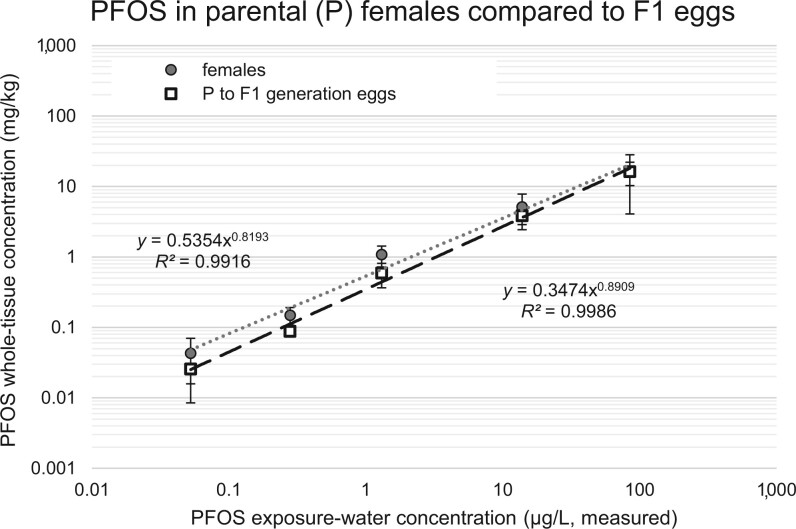
Investigation of perfluorooctane sulfonic acid (PFOS) maternal transfer from parental (P) generation females exposed through 180 days postfertilization (dpf) into first filial (F1) generation eggs, where the eggs were spawned into clean water and collected after 7–8 hours. Values represent PFOS accumulation in zebrafish whole-body tissue wet weights for the female fish and PFOS retained in whole egg tissues (F1 generation eggs were never exposed to PFOS-containing exposure medium). Statistical comparison of female body burdens and egg burdens indicated a statistically significant difference (*p *= .017); however, there were no significant differences observed between females and eggs at any given exposure concentration in Holm-Sidak pairwise comparisons tests (*p *> .05).

Perfluorooctane sulfonic acid accumulated in zebrafish eggs with burdens that tended to match the concentrations observed in the maternal whole-body tissues (Figure 5). The PFOS burdens in both the maternal tissue and eggs increased significantly with increasing PFOS exposure concentrations and a significant difference was observed between PFOS concentrations in maternal whole-body tissues and eggs (*p *= .017, f = 6.74). However, no significant differences were observed in the PFOS burdens between the maternal tissue and eggs at each PFOS exposure concentration in pairwise comparison tests (Figure 5).

### Fidelity of the toxicokinetic model

**Figure 6.**
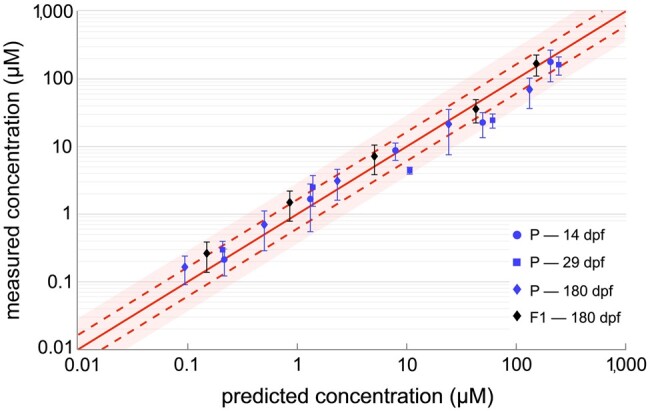
Fidelity of toxicokinetic model predictions in parental (P) and first filial (F1) generations of zebrafish exposed continuously for varying days postfertilization (dpf). Dotted lines represent *SD* (=1.64 fold) about the line of equality. The shaded region represents twofold above and halffold below the line of equality.

Figure 6 illustrates the fidelity of our model (Table 2) fit to data reported herein. To a reasonable accuracy, *Equation 6* quantitatively reproduces PFOS body burden measurements in both the P generation (blue symbols) at 14, 29, and 180 dpf and F1 generation (black symbols) at 180 dpf durations of the exposure data. For use in a risk assessment, toxicokinetic model predictions should fall within a factor of 2 for the available data, as reported by the International Programme on Chemical Safety (World Health Organization [WHO], 2010). The model estimates of Figure 6 vary in the direction perpendicular to the line of equality (solid red) by 1.64-fold above and .61-fold below it (dotted lines), whereas the shaded area represents a region bounded twofold above and .5-fold below it. Thus, estimates from our toxicokinetic model fall within the acceptable margin of error for application to PFOS risk assessment for larval, juvenile, and combined sexes of adult zebrafish.

**Table 2.** Parameter values included in the toxicokinetic model.

Symbol	Value	Unit	Description
T	62.23	hr	Characteristic hatching time as hours postfertilization (hpf), scale parameter of the log-logistic distribution function
α	19.25	—	Hatching exponent, shape parameter of the log-logistic distribution function
$V_{\infty }$	.51148	mm^3^	Asymptote for the volume of adult zebrafish
τ	4282.1	hr	Characteristic growth time of zebrafish growth and development
ρ	1	$\frac{g}{{\text{mm}}^{3}}$	Unit density of water
σ	.00265	$\frac{g}{{\text{mm}}^{2}}$	Scale parameter for the allometric relationship between gill surface area and mass of adult fish species
K	1.17	*µ*M	Scale parameter for aqueous exposure concentrations
k	1.604	$\frac{\mu \text{mol}}{{\text{mm}}^{2}\;\text{hr}}$	Perfluorooctane sulfonic acid (PFOS) mass uptake per unit exchange surface area and time
$k_{d}$	.00151	$\frac{L}{{\text{mm}}^{2}\;\text{hr}}$	PFOS elimination constant in units of tissue volume per unit exchange surface area and time

## Discussion

### PFOS accumulation in zebrafish

This study provides the first observations of PFOS accumulation in multiple zebrafish life stages exposed continuously through two generations. Accumulation of PFOS in zebrafish whole-body tissues increased with increasing PFOS concentrations in exposure water, approaching an apparent steady state by the first time point investigated (14 dpf). Previous investigations of PFOS accumulation in zebrafish embryos/larvae indicate rapid accumulation versus time (Huang et al., 2010; Vogs et al., 2019; Zou et al., 2021). The study by Vogs et al. (2019) identified that PFOS accumulation in zebrafish embryos approached steady state within 120 hr of exposure to PFOS at 20 and 40 µg/L, whereas an order of magnitude higher exposure (380 µg/L) resulted in continuously increased PFOS accumulation through the 120 hr exposure time point. In that study, PFOS kinetics were reported for uptake into zebrafish embryos, wherein slow uptake was observed through approximately the first 48 hr of exposure while the chorion was intact, followed by more rapid uptake in the 48 hr after the embryos had hatched from the chorion shell. Correspondingly, Mylroie et al. (2021) observed that the presence of the chorion delayed PFOS exposure-related morphological effects and mortality in zebrafish embryos relative to embryos that had been dechorionated; however, the effects of PFOS exposures on mortality were equivalent among chorionated and dechorionated zebrafish by 120 hr of exposure. Although the statistical significance of any biphasic character in the embryo PFOS uptake data has not been established (Warner et al., 2022), the general observations of Vogs et al. (2019) and Mylroie et al. (2021), when taken together, suggest that a potentially lower PFOS accumulation by the chorion serves to minimally delay the ultimately rapid accumulation and effects of PFOS in zebrafish embryos once hatched.

Remarkably, the results of this study indicated that PFOS body burdens remain relatively constant through time in zebrafish from 14 dpf, to 29 dpf, and ultimately through 180 dpf, in both the P and F1 generations despite dramatic developmental changes in the fish from larval stages through adulthood. Toxicokinetic modeling identified that PFOS accumulation in fish tissues is fundamentally dynamic; the pace of whole-organism PFOS accumulation was approximately matched by the rate of zebrafish growth during the developmental period and beyond into adults, which continue to grow up to and beyond the sampling period. On average, PFOS body burdens remained constant (statistically identical) from larval to adult stages (Figure 2). Separate investigations of male and female body burdens indicated approximately twofold higher PFOS accumulation in male fish relative to females in both the P and F1 generations exposed continuously through 180 dpf (Figure 3). Wang et al. (2011) also found significantly greater PFOS accumulation in male versus female fish after continuous exposure for 5 months that resulted in increased relative survivorship of females. In our study, zebrafish were undergoing weekly breeding trials from 124 dpf through 180 dpf, which may have served as a potential PFOS depuration pathway for females relative to males. This subject is discussed in more detail in the *Maternal transfer* section below.

### PFOS bioconcentration in zebrafish

Perfluorooctane sulfonic acid dissolved in exposure water bioconcentrated in zebrafish whole-body tissues resulting in BCF values ranging from 276 to 2,187 L/kg with an overall mean across all observations of 960, a median of 934, and *SD* ± 443 (Figure 4). A literature review of PFAS accumulation and bioconcentration in aquatic species by Burkhard (2021) established the median PFOS BCF for teleost fish at 1,023 with *SD* ± 224 based on 21 total observations. Tal and Vogs (2021) summarized BCF value observations for PFOS in zebrafish gathered from five published studies investigating multiple life stages and a nearly four order of magnitude range in PFOS exposure concentrations. In that study, BCFs for zebrafish whole-body tissues ranged from 82 to 5,400. Across these observations, the BCFs decreased with increasing PFOS concentrations in exposure water, a result that was also observed in our study regardless of zebrafish life stage, sex, generation, or exposure duration (Figure 4). In Tal and Vogs (2021), whole-body tissue BCFs for PFOS tended to be similar between adult and larval zebrafish, although the adult PFOS exposures were administered at lower concentrations relative to the studies with larvae. In our study, the PFOS exposure concentrations were equivalent for the larval, juvenile, and adult zebrafish, generally within a factor of approximately 2 (Figure 2B), whereas the continuous exposure duration increased through time (i.e. 14, 29, or 180 day exposures), where BCFs only varied by approximately a factor of two comparing the subadult zebrafish (14 and 29 dpf) versus the adults at 180 dpf (Figure 4).

The trend of decreasing BCFs with increasing exposure concentrations has been observed for a broad range of aquatic organisms and across various PFAS (Burkhard, 2021), and specifically for PFOS in the common carp (Inoue et al., 2012) and zebrafish (Chen et al., 2016; Han et al., 2021). Various hypotheses have been posited to explain this observation, including limited transport surfaces affecting bioaccumulation dynamics and competition for molecular binding sites for PFOS such as binding to serum proteins (Zhong et al., 2019). The mechanisms underlying PFAS bioaccumulation are complex; where affinity for and transport through phospholipids is broadly recognized, binding to albumin protein has been identified as a systemic transport mechanism via blood circulation, partitioning to liver-fatty acid binding protein can serve as a PFAS sink, and active transport by organic anion transporters can concentrate PFAS in renal tissue (Ng & Hungerbühler 2014). Evidence of differential partitioning of PFOS within fish has been demonstrated, where increased BCFs have been observed in blood and liver relative to whole fish in both zebrafish and rainbow trout (Chen et al., 2016; Martin et al., 2003).

A decreased BCF at higher exposure concentrations can also be reproduced by our toxicokinetic model. Dependence of the uptake flux (first term on the right-hand side of *Equation 5*) is nonlinear in the exposure concentration, which has been directly translated from the embryo uptake model of Warner et al. (2022). Because our experiments suggest that PFOS body burden is approximately independent of the exposure duration beyond 14 dpf (Table 1), the uptake and elimination rates of *Equation 5* are balanced on this shorter timescale (with organism growth a longer timescale). If we apply this condition to *Equation 5*, we find that whole-body concentrations are proportional to the term $\frac{C_{\;\text{exp}\;}}{K+C_{\;\text{exp}\;}}$, which means that $\text{BCF}\propto \frac{1}{K+C_{\;\text{exp}\;}}$. Our model thus predicts a decrease in BCF near $C_{\;\text{exp}\;}\approx 585\;$µg/L. This prediction is quantitatively inaccurate, as trends from Figure 4 show a transition from higher to lower BCFs between 1 and 10 µg/L. This discrepancy could mean that the aqueous exposure scale parameter, K, of our model is overestimated by about one to two orders of magnitude. This is plausible, because this parameter lumps together contributions from a wide range of biological processes associated with tissue uptake (e.g., passive and active transport across cell membranes, binding to transporter chaperones), thus making its value sensitive to the myriad of tissue binding/translocation mechanisms mentioned above.

Finally, increased BCF values for PFOS in male zebrafish relative to females (Figure 4) has been observed in other long-term exposures in zebrafish (Wang et al., 2011). An analogous occurrence of increased PFOS bioaccumulation has been observed in humans, where PFOS burdens in male blood serum were significantly higher than females in an investigation of 3,802 Australian residents (Kärrman et al., 2006). Mechanistic differences in PFAS toxicokinetics among sexes have been described by Ng and Hungerbühler (2013), where increased expression of organic anion transporters by male rats may more efficiently recapture perfluorooctanoic acid (PFOA) from urine and remain within systemic circulation (Weaver et al., 2010) and observations of nearly tenfold slower elimination rates of PFOA from blood plasma of male fathead minnows was connected to androgen metabolism (Lee & Schultz, 2010). Connecting PFOS body burdens from the present study to the associated ecotoxicological effects reported in Gust et al. (2024), increased body burdens in male fish corresponded with an increased number of significant impacts on growth (weights and lengths) relative to females. Overall, the observation of increased PFAS accumulation and BCFs in males relative to females in multiple species requires additional attention during risk characterization for specific sexes.

### Maternal transfer of PFOS to eggs

A separate potential route of PFAS elimination available to females is transfer to eggs/offspring. Observations by Conard et al. (2022) and Wang et al. (2023) indicated the potential for maternal offloading of PFOS to eggs in wild caught salmonid species and catfish, respectively. In addition, Sharpe et al. (2010) observed maternal transfer of PFOS to eggs in zebrafish where PFOS concentrations were 1.6-fold higher in eggs versus maternal tissue representing an estimated 10% maternal PFOS body burden transfer into eggs. In our study, no significant difference was observed in the concentration of PFOS residues in eggs relative to the whole body burdens measured in the maternal females (Figure 5). As a methodological note for our study, breeding trials were conducted in control water (no PFOS treatment) and eggs held for 7–8 hr in control conditions prior to fixation. Previous observations and estimates of PFOS elimination in zebrafish embryos suggest slow PFOS loss rates from tissue (Huang et al., 2010; Zou et al., 2021), where the holding time of eggs investigated in our study was not expected to have resulted in appreciable loss of PFOS prior to tissue fixation. Regardless of the pathway by which maternal transfer of PFOS to eggs occurs, maternally transferred PFOS has been attributed to increased zebrafish larval lethality (Wang et al., 2011). Improved understanding of how PFAS maternal transfer processes operate in fish has the potential to refine toxicodynamics models for the maternal fish as well as establish PFAS burdens passed to eggs, a process that may ultimately inform population-level sustainability estimates.

## Conclusion

Accumulation of PFOS in zebrafish appears to reach a whole-body steady-state concentration within 14 days of exposure and likely even earlier, based on previous studies (Vogs et al., 2019), and remain constant through 180 days of continuous PFOS exposure and even through a subsequent complete F1 generation exposed for 180 days. The median BCF of 934 L/kg observed in zebrafish exposed to PFOS in our study closely corresponds to the median BCF of 1,023 L/kg calculated from a literature review by Burkhard (2021) for teleost fish. Further, our toxicokinetics model predicts a BCF value of 908.8 L/kg for PFOS exposure concentrations below approximately 1.17 µM. Based on our experimental observations, variance in BCFs was related to PFOS exposure concentration, where higher exposure levels resulted in lower BCFs and also varied with sex, where males had BCFs approximately twofold greater than females. Maternal transfer of PFOS to eggs was confirmed in the this study, but similar tissue residues among maternal females and their eggs suggests that females did not depurate PFOS burdens via transfer to offspring. The toxicokinetic model developed herein reliably reproduced PFOS whole-body burdens (data to within 1.64-fold of predicted values) in zebrafish larvae, juveniles, and combined sexes of adults exposed continuously through 180 dpf and through 180 dpf in the subsequent F1 generation. This model provides novel utility for prediction of PFOS accumulation in response to environmentally-relevant exposures and is therefore suitable for use in risk assessments of acute, chronic, and multigenerational exposure conditions. Finally, research is needed to experimentally determine the physiological/mechanistic processes causing increased PFOS body burdens in adult male zebrafish relative to females to develop future sex-specific toxicokinetic models that can enable high-fidelity risk characterization for the more sensitive sex.

## Supplementary Material

vgae033_Supplementary_Data

## Data Availability

All data are available on request to the corresponding author (kurt.a.gust@usace.army.mil).
