# Supplementary material for: Bioconcentration, maternal transfer, and toxicokinetics of PFOS in a multi-generational zebrafish exposure
Source: Environ Toxicol Chem. 2025 Jan 6;44(1):207–19. doi: 10.1093/etojnl/vgae033 (PMC11790210; doi:10.1093/etojnl/vgae033)
Supplement: vgae033_Supplementary_Data [file vgae033_supplementary_data.docx]

***Supplemental Materials:***

**Bioconcentration, Maternal Transfer, and Toxicokinetics of PFOS in a Multi-Generational Zebrafish Exposure**

Kurt A. Gust^1^*, Ashley N. Kimble^1^, J. Erik Mylroie^1^, Michael L. Mayo^1^, Mitch S. Wilbanks^1^, Catherine S. C. Steward^2^, Kacy A. Chapman^1^, Guilherme R. Lotufo^1^, Natalia Garcia-Reyero^1^, David W. Moore^1^

Affiliations:

^1^US Army, Engineer Research and Development Center, Environmental Laboratory, Vicksburg, MS

^2^Bennett Aerospace, Cary, NC

*Corresponding author: kurt.a.gust@usace.army.mil

*Running Title:* PFOS Bioconcentration in Multi-Gen Zebrafish Exposure

Supplemental Table S1. The cumulative water quality measurements for the P generation larvae sampled at 14- and 29-days post fertilization (dpf) for the static portion of the exposure (0-14 dpf) and the flow-through portion of the exposure (15-29 dpf). Water quality measurements show the mean, maximum, and minimum measurements from the static and flow-through portions of the exposure.

^1^Conductivity values during the static exposure were higher than those for the flow-through exposures to as E2 media (Varga et al. 2016), which has a higher conductivity than the flow-through water, was used for solutions during the static phase of the exposure.

^2^Temperature did not vary among treatments during exposures.

Supplemental Table S2. Table reproduced from Gust et al. (2024). The cumulative water quality measurements for the P and F1 generations. Water quality measurements show the mean, maximum, and minimum measurements from days 6 – 180.

^1^Conductivity values during the static exposure were higher than those for the flow-through exposures to improve the ability for rotifers to stay in the water column and facilitate zebrafish larval feeding.

^2^Temperature did not vary among treatments during exposures.

^3^Measurements for alkalinity and hardness taken at a single timepoint at the start of the P generation exposure for background values. Measurements are a mean of values taken from two random replicates from each of the treatment groups.

**Literature Cited**

Gust, K.A., Mylroie, J.E., Kimble, A.N., Wilbanks, M.S., Steward, C.S.C., Chapman, K.A., Jensen, K.M., Kennedy, A.J., Krupa, P.M., Waisner, S.A., Pandelides, Z., Garcia-Reyero, N., Erickson, R.J., Ankley, G.T., Conder, J., Moore, D.W., 2024. Survival, Growth, and Reproduction Responses in a Three-Generation Exposure of the Zebrafish (Danio rerio) to Perfluorooctane Sulfonate. Environ. Toxicol. Chem. 43, 115-131.

Varga, Z. M. (2016). Aquaculture, husbandry, and shipping at the Zebrafish International Resource Center. In W. H. Detrich, M. Westerfield & Z. I. Leonard (Eds.), *The zebrafish: Genetics, genomics, and transcriptomics* (Vol. **135**, *Methods in cell biology* 4th ed., pp. 509–534). Academic Press.
